# Phylogeny and taxonomy of Haloclavidae (Verrill, 1899) with a redescription of the parasitic, burrowing sea anemone, *Peachia chilensis* Carlgren, 1931

**DOI:** 10.1371/journal.pone.0266283

**Published:** 2022-09-16

**Authors:** Natalie Hamilton, Luciana C. Gusmão, Takato Izumi, Estefanía Rodríguez, Nicholas W. L. Yap, Marymegan Daly

**Affiliations:** 1 Department of Rangeland, Wildlife, and Fisheries Management, Texas A&M University, College Station, TX, United States of America; 2 Division of Invertebrate Zoology, American Museum of Natural History, New York, NY, United States of America; 3 Molecular Invertebrate Systematics and Ecology Laboratory, Department of Biology, Chemistry, and Marine Sciences, Faculty of Science, University of the Ryukyus, Nishihara, Okinawa, Japan; 4 Tropical Marine Science Institute, National University of Singapore, Singapore, Singapore; 5 Department of Evolution, Ecology, and Organismal Biology, The Ohio State University, Columbus, OH, United States of America; Sao Paulo State University (UNESP/FCL/Assis), BRAZIL

## Abstract

Haloclavidae Verrill, 1899 is a family of burrowing sea anemones grouped within the superfamily Actinioidea (Rafinesque, 1815). Currently, it includes 30 species in 10 genera. Characters given for this family in descriptions of its taxa have not been consistent, with numerous exceptions to the expectations of the familial diagnosis. Previous phylogenetic analyses have shown that Haloclavidae is potentially a polyphyletic group, but resolution of relationships of the few representatives of Haloclavidae included in analyses has been problematic. Here we address questions of monophyly and affinity of Haloclavidae using three mitochondrial and two nuclear markers. We assess the monophyly of Haloclavidae in the context of all major lineages of Actiniaria Hertwig, 1882, emphasizing diversity of superfamily Actinioidea. We use parsimony-based character optimization to interpret the distribution of key traits in the superfamily. We find that Haloclavidae is not monophyletic and propose two new families, Peachiidae fam. nov. and Harenactidae fam. nov., while also retaining some species in the family Haloclavidae, so that taxonomy better reflects relationships and diversity of the group. In addition, we redescribe a species within the newly created Peachiidae, *Peachia chilensis* Carlgren, 1931. We use recent larval samples obtained in Antofagasta, Chile, and the histological slides from the original description to redescribe *P*. *chilensis*, to provide a complete account of cnidae, external, and internal morphology. Finally, we compare *P*. *chilensis* to other burrowing anemones found in Chile and provide an understanding of the genus *Peachia* that reflects recent phylogenetic perspective on diversity of anemones previously assigned to family Haloclavidae.

## Introduction

Sea anemones (order Actiniaria Hertwig, 1882 [1]) are a diverse group of animals in the cnidarian subclass Hexacorallia Haeckel, 1896 [2]. They are sessile, benthic marine invertebrates that lack a skeleton and exist in their polyp form as adults [3]. Anemones show high levels of morphological convergence with some morphological characteristics also being repeatedly lost throughout their evolutionary history [4, 5]. Many superfamilies, families, and genera have been grouped based on the absence of features rather than synapomorphies [6]. DNA-based phylogenetic analyses have repeatedly revealed the inadequacy of morphology-based classifications of this order [4, 6].

Burrowing sea anemones were historically grouped into the Infraorder Athenaria Carlgren, 1899 [7] based on their shared lack of basilar muscles [3]. However, this classification does not align with the results of DNA-based phylogenies. Athenaria has been revealed as polyphyletic, reflecting a repeated loss of basilar musculature rather than a single loss of this trait [4–6, 8–11]. At present, phylogenetic analyses find burrowing anemones in superfamilies Edwardsioidea (Andres, 1881) [12], Actinioidea (Rafinesque, 1815) [13], Actinostoloidea Carlgren, 1932 [14], and Metridioidea (Carlgren, 1893) [4, 5, 15]. All members of Edwardsioidea are burrowers, but within the other superfamilies, the burrowing species do not form monophyletic groups.

Haloclavidae (Verrill, 1899) [16] is a family of burrowing sea anemones within superfamily Actinioidea. Based on their phylogenetic placement, it is inferred that members of Haloclavidae have lost the endodermal marginal sphincter and basilar musculature that are present in many other members of Actinioidea [4]. Haloclavidae includes 10 genera and 30 species [17]. Broad-scale phylogenetic analyses have revealed that Haloclavidae is not a monophyletic group, but resolution of relationships for the few representatives included has been problematic [4, 5]. For example, the haloclavids *Stephanthus* Rodríguez & López-González, 2003 [18] and *Harenactis* Torrey, 1902 [19] are consistently recovered as sister genera, but their relationship to other Haloclavidae is inconsistent across analyses [4, 5]. The relationship of the type genus, *Haloclava* Verrill, 1899 [16], to other genera is also not resolved in a consistent way across analyses or data sets. Because the characteristics used to group species within Haloclavidae are potentially convergent traits that allow for burrowing, these findings are not surprising.

The current circumscription of Haloclavidae derives from Barragán et al. [20] and the diagnosis slightly expanded from the original description by Verrill [16] and subsequent revision by Carlgren [2]. According to Barragán et al. [20], the diagnostic features of this family are a vermiform body with a physa-like aboral end or pedal disc, a column not (or only weakly) divisible into distinct regions, generally short tentacles, and a single, strong siphonoglyph. The characters used to diagnose members of this family are each also characteristic of other taxa and have a high range of variability within Haloclavidae, with numerous exceptions to the rules identified by previous authors, including Verrill [16] or Carlgren [18, 21]. Due to their burrowing, coloration, and size, these animals are hard to find alive and the anatomy and function of many features is poorly described. Several genera and species of Haloclavidae have been described based on a single specimen. Describing species, and especially erecting new genera, based on a single specimen can cause taxonomic problems because variation in traits cannot be captured in a single individual.

The conchula is an important feature for taxonomy in some genera of Haloclavidae, but its function and connection to other features is not well understood [9, 22]. The conchula is a lobate protrusion of the siphonoglyph onto the oral disk that is hypothesized to either help with feeding or water flow into the coelenteron [22]. The siphonoglyph is a ciliated groove in the actinopharynx, and in animals without a conchula, it funnels water into the gastrovascular cavity of the sea anemone. *Peachia* Gosse, 1855 [23] was the first genus in the family for which the conchula was used as a diagnostic characteristic; subsequently described taxa having a conchula are *Metapeachia* Carlgren, 1943 [24], *Synpeachia* Yap, Fautin, Ramos, & Tan, 2014 [25], *Antennapeachia* Izumi, Yanagi & Fujita, 2016 [26], and *Tenactis* Barragán, Sánchez & Rodríguez, 2018 [20]. This feature is not uniform in morphology across these genera and it is also present in other actiniarians outside of Haloclavidae (e.g. *Actinoporus* Duchassaing, 1850 [27]).

Other than these genera having a conchula, the genera within Haloclavidae are not linked by clear morphological traits other than a vermiform shape and a single, strong siphonoglyph. The type species of *Haloclava*, *Haloclava producta* (Stimpson, 1856) [28], has acrospheres (nematocyst batteries forming a nodule at a tentacle apex), a characteristic that is reported in species across Hexacorallia and also present in species of the haloclavid genus *Anemonactis* Andres, 1881 [12]. *Haloclava* and *Anemonactis* are distinguished by differences in column papillae and in the presence/absence of cinclides, openings in the column of the animal. In the literature, there is no report of *Haloclava* having cinclides. Cinclides can be difficult to see depending upon the contraction of the animal at the time of fixation, and it is possible that members of *Haloclava* do indeed have this trait [9, 29]. The genera *Harenactis* and *Stephanthus* have been resolved as a clade independent of other haloclavid anemones, but this relationship has not been proposed previously or explored, and defies easy categorization based on anatomy [4, 5].

We construct a DNA-based phylogeny to understand relationships among the genera assigned to Haloclavidae. Because the diagnosis of this family has been modified several times within the last decade to accommodate new taxa [e.g. 9, 20, 28], the monophyly of the group should be reexamined to determine if these modifications to the family are necessary or accurate. The morphological basis for boundaries between Haloclavidae and other actinioidean families is unclear. Further, genera of Haloclavidae are species-poor and known from relatively few specimens, which contributes to difficulties in understanding taxonomic affinities.

We conduct a phylogenetic analysis that includes 192 species across five superfamilies, so that we can test 1) the monophyly of the Haloclavidae and identify its closest known relatives; 2) identify relationships among the “*Peachia*-like” anemones and 3) evaluate the pattern of change for key features. We use three mitochondrial (COIII, 12S rDNA, 16S rDNA) and two nuclear (18S rDNA, 28S rDNA) markers in order to accomplish these goals. The scope of this analysis is the largest to date for Actinioidea in terms of the number of genera or species included. We aim to understand how taxonomically important features like conchula and acrospheres change across this phylogeny so that we can understand whether these are more important than attributes like tentacle number or anatomy, in terms of providing grouping information. This phylogeny guides a revision of the composition and diagnosis of Haloclavidae, with the primary goal of recognizing diagnosable units that form monophyletic groups.

To further strengthen this primary objective and the taxonomy of each monophyletic group that resulted from our analyses, we revise and expand the diagnosis of *Peachia*, through a redescription of *P*. *chilensis* using a significant number of fresh larval specimens that we have collected, and comparisons to its type material. Like most species of *Peachia*, *P*. *chilensis* are parasitic on medusae (jellyfish) during their larval stage. Because the anemones are more easily discovered when they are attached to jellyfish than when they are buried in substrate, 5 of the 11 species of *Peachia* species are described from a single adult specimen and two species are described from only the larval state. Currently, species described from the larval stage are treated as valid [3], but the affinity and adult anatomy of these remains unclear.

## Materials and methods

### Taxonomic sampling and data collection

We include 11 representatives of Haloclavidae: nine nominal species representing seven genera within the family, with new DNA sequence data for eight individuals within the family (S1 Table). Sequences from GenBank were included as appropriate (S1 Table). To test the monophyly of Haloclavidae, we included as many taxa as possible from the family Haloclavidae and related taxa in Actinioidea. We use the zoanthid *Savalia savaglia* (Bertoloni 1819 [30]) as our chosen outgroup as it has been used previously in large scale anthozoan phylogenies [31]. We only included taxa for which we were able to amplify at least three of the five markers and thus have analyzed a total of 1,002 sequences for 226 terminal taxa (192 unique species). Specimens were collected by hand, during SCUBA dives, or via trawls. Additionally, 15 specimens identified as the parasitic sea anemone *Peachia chilensis* were collected in June 2010 off the coast of Antofagasta, Chile. The sampling did not involve endangered or protected species.

Total genomic DNA was isolated from tentacle tissue, column tissue, or whole animals (in the case of *Peachia quinquecapitata* McMurrich, 1913 [32]) using DNeasy Blood and Tissue Kits (QIAGEN Inc.) and stored at -20°C. Genomic DNA was amplified using published primers and standard techniques [6, 31, 32]. Three mitochondrial (COIII, 12S rDNA, and 16S rDNA) and two nuclear (18S rDNA and 28S rDNA) markers were targeted for phylogenetic reconstruction as these loci have been widely used in actiniarian phylogenetic studies to answer questions like those we pose [4–6, 31]. PCR reactions were carried out in 25 μL volumes using Illustra-TM puReTaq Ready-To-Go PCR beads (GE Healthcare).

Samples were cycle sequenced in both directions at TACGen DNA Sequencing in Richmond, CA, USA. Consensus sequences were generated in Geneious 7.1.9 [33]. Once assembled, contigs were queried against the nucleotide database of NCBI using BLAST in order to identify possible contaminants. All sequences have been deposited in GenBank, with new sequences in bold (S1 Table).

### Phylogenetic analyses

Sequences for each marker were aligned in MUSCLE [34] using the default parameters and implemented through Geneious v. 2021.0.1 [33]. Sequences were concatenated into mitochondrial (COIII, 12S rDNA, and 16S rDNA), nuclear (18S rDNA and 28S rDNA), and “all-loci” data sets. The concatenated alignments are used for all phylogenetic reconstructions. All combined matrices were submitted to PartitionFinder [35] under the corrected Akaike information criterion [36] to test the partitioning schemes. PartitionFinder was implemented through CIPRES Science Gateway [37]. All analyses described below were conducted on three datasets: the mitochondrial dataset, the nuclear dataset, and the all-loci concatenated data set (mitochondrial and nuclear combined).

Maximum likelihood analyses were run in IQ-TREE 2.1.1 [38] using the partition schema and substitution models discovered by PartitionFinder. The COIII alignment was partitioned by codon position. We assessed support by running 1000 ultrafast bootstrap replicates. Analyses performed using MrBayes v. 3.2.7 [39] were implemented through CIPRES Science Gateway. Run parameters can be found in S2 Table. Alignments were analyzed separately and in combination using parsimony as implemented in PAUP v. 4.0a [40]. Gaps were treated as ambiguous rather than as a fifth state. The datasets were subjected to 1000 bootstrap replicates to assess branch support on the consensus tree.

### Ancestral state reconstruction

Maximum parsimony ancestral state reconstructions were performed using Mesquite v. 3.51 [41] for four key morphological characters (basilar muscles, conchula, acrospheres, marginal sphincter muscle) (S4 Table) at all internal nodes of the phylogenetic tree derived from the maximum likelihood analysis. Nodes with <50 support were not reported for the reconstructions.

### Morphological investigations

Larval *Peachia chilensis* were removed from their jellyfish hosts by hand and preserved in 99% ethanol. These preserved specimens were examined whole, one was examined via micro-CT scanner, two were dissected, and four were used in histological preparations. The specimen examined through micro-CT scanning followed the protocol outlined in [42] with some modifications: the final resolution was set at 19.41 μm/voxel and the exposure time for the detector was 750.182 ms. Serial histological sections 5–8 mm thick were stained with an Azocarmine triple-stain protocol modified from Humason [43].

The cnidom of *P*. *chilensis* was assessed from seven individuals. Squash preparations were mounted from preserved pieces of tentacles, column, actinopharynx, and mesenteries. Un-fired capsules of each type of cnida were haphazardly chosen, measured, and photographed using DIC at 1000x total magnification. The mean and standard deviation of the measured capsules offer an understanding of their size distribution but are not statistically significant. Cnida nomenclature follows Gusmão et al. [42], which differentiates “basitrichs” from “*b*-mastigophores” (see [44, 45]) and follows Schmidt’s [46, 47] suggestions about variation in “rhabdoids.” All specimens and preparations are deposited at the American Museum of Natural History (AMNH).

We compared our material to slides made from paratypes deposited at The Biologiska Museet in Lund, Sweden (MZLU). Paratype specimens were unavailable to be loaned. Taxonomic results are reported in the S1 Appendix.

## Results

### Markers and congruence

The sequences ranged in length from 67–3379 and contained 21–45% parsimony informative sites after alignment (S2 Table). The nuclear dataset did not include *Anemonactis minuta* (Wassilief, 1908 [48]) due to lack of high-quality sequence data for both 18S rDNA and 28S rDNA markers. In general, nuclear markers were longer than mitochondrial markers and were more variable in length, thus requiring more gaps to align sequences. Our Bayesian inference method (MrBayes) failed to converge in all cases (S3 Table), but we report findings below. All analyses and datasets found the included members of Haloclavidae to be within Actinioidea and recovered relationships among suborders and superfamilies found in other studies (e.g., [4]). No analysis or dataset found monophyly of Haloclavidae (Table 1).

**Table 1:** Resolution of key relationships across each data set.

Relationship	Parsimony			Likelihood			Bayesian		
Dataset	all	mt	nu	all	mt	nu	all	mt	nu
*Anemonactis* + *Haloclava* sp. + *Harenactis* + *Stephanthus* + *Antennapeachia* + *Metapeachia* + *Peachia* + *Synpeachia* (Haloclavidae monophyletic)	N	N	N	N	N	N	N	N	N
*Anemonactis* + *Haloclava* sp. (Clade 1)	Y	**Y**	-	**Y**	Y	-	N	**Y**	-
*Haloclava* sp. + *Haloclava producta*	N	N	N	N	N	N	N	N	N
*Harenactis* + *Stephanthus* (Clade 2)	**Y**	**Y**	N	**Y**	**Y**	N	N	**Y**	N
*Antennapeachia* + *Metapeachia* + *Peachia* + *Synpeachia* (Clade 3)	N	N	N	**Y**	Y	N	**Y**	**Y**	N
*Peachia cylindrica* + *P*. *quinquicapitata*	**Y**	Y	N	**Y**	**Y**	N	**Y**	**Y**	N
*Metapeachia* + *Synpeachia*	**Y**	U	Y	**Y**	**Y**	Y	**Y**	U	Y
*Peachia* + *Metapeachia* + *Synpeachia*	**Y**	Y	N	**Y**	**Y**	U	**Y**	**Y**	N
*Antennapeachia* + *Anemonactis* + *Haloclava* sp.	Y	U	-	N	N	-	N	N	-

For each possible grouping and analysis, Y indicates that the listed taxa are sisters, N indicates that they are not sisters, U indicates that they are in the same clade but unresolved in terms of their sister group relationship. “-” indicates that the relationship was not tested. Groupings with bootstrap values >85 are indicated in bold. Abbreviations: All = all-loci concatenated dataset, mt = mitochondrial dataset, nu = nuclear dataset.

### Model based analyses

**Fig 1:**
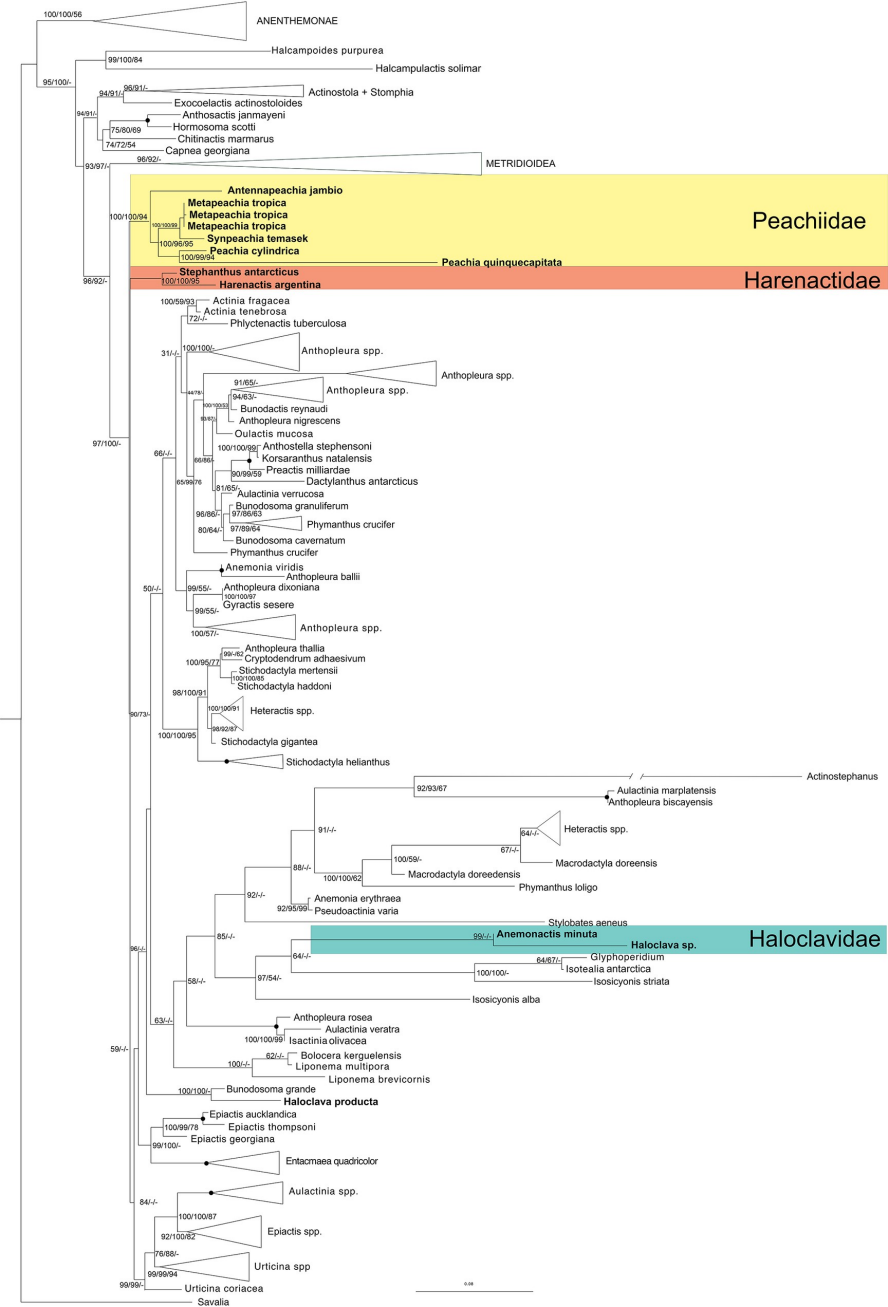
Phylogenetic reconstruction of Actiniaria. Tree resulting from the maximum likelihood analysis of the all-loci dataset (12S, 16S, 18S, 28S, and CO3). Numbers on branches are bootstrap resampling values for ML and parsimony, respectively, expressed as a percent, followed by posterior probabilities (multiplied by 100) for legibility. Filled circles indicate nodes with 100% support for all inferences.

In the tree of highest likelihood for the all-loci dataset, Haloclavidae is split into three well-supported clades within Actinioidea (Fig 1). Clade 1 consists of *Haloclava* sp. and *Anemonactis minuta*. This clade is not associated with other genera of Haloclavidae but is instead sister to a clade that contains the Antarctic actiniids *Glyphoperidium bursa* Roule, 1909 [49] and *Isotealia antarctica* Carlgren, 1899 [7]. *Haloclava producta* is not contained within Clade 1 but resolves as sister to *Bunodosoma grande* (Verrill 1869) [50] at full support. Clade 2 consists of *Harenactis argentina* Lauretta, Rodríguez & Penchaszadeh, 2011 [29] and *Stephanthus antarcticus* Rodríguez, & López-González, 2003 [18]. Clade 3 consists of *Antennapeachia*, *Metapeachia*, *Peachia*, and *Synpeachia*. Within Clade 3, *Antennapeachia jambio* Izumi, Fujita & Yanagi, 2017 [51] is sister to the other members of the group and the two *Peachia* species are sister to a clade comprised of *Synpeachia temasek* Yap, Fautin, Ramos & Tan, 2014 [25] and *Metapeachia tropica* (Panikkar, 1938) [52]. Clade 3 is sister to the larger group of actinioideans, which includes both Clade 1 and Clade 2. In general, branch lengths for the included species of Haloclavidae are similar to those of the other actinioidean species, with the notable exception of *P*. *quinquecapitata*, which has a relatively long branch.

The tree of highest likelihood for the mitochondrial dataset recovers the same three clades for Haloclavidae as in the all-loci analyses, but each has different sister group relationships within Actiniodea. Clade 2 is sister to a group containing deep-sea and polar actiniids (e.g. *Isosicyonis* Carlgren, 1927 [53], *Liponema* Hertwig, 1882 [1], etc) plus tropical actiniids (e.g *Entacmaea quadricolor* (Leuckart in Rüppell & Leuckart, 1828) [54], *Actinostephanus haeckeli* Kwietniewski, 1897 [55], and *Stylobates aeneus* Dall, 1903 [56]) but this relationship has low support. In the likelihood analysis of the nuclear markers Haloclavidae does not resolve into the three clades that were seen in the other datasets. *Haloclava* sp. is sister to *Isosicyonis alba* (Studer, 1879) [57]. All of the species near this *Haloclava*/*I*. *alba* clade have particularly long branches, compared to the typical branch length for this tree. *Haloclava producta*, *Bunodosoma grande*, and *Antennapeachia jambio* form a clade in which *An*. *jambio* is strongly supported as sister to the other two species. The branch length for *An*. *jambio* is longer than the branch between *Bd*. *grande* and *Ha*. *producta*. *Stephanthus antarcticus* is sister to *Epiactis georgiana* Carlgren, 1927 [53] with high support. *Harenactis* resolves near a larger clade that contains members of *Anthopleura* Duchassaing de Fonbressin & Michelotti, 1860 [58] and other actiniid species. *Metapeachia* is sister to *Synpeachia temasek* and *Peachia quinquecapitata; Peachia cylindrica* is not within this clade, being instead strongly supported as sister to the metridioidean deep-sea species *Jasonactis erythraios* (Zelnio, Rodríguez & Daly 2009) [59].

### Bayesian

In the tree with the highest posterior probabilities for the all-loci dataset, Haloclavidae is split into two major clades corresponding to Clade 2 and Clade 3 of the likelihood analyses. Due to failure to converge, the topology of this tree is not as well-resolved as in the maximum likelihood and parsimony trees. *Haloclava producta* is sister to *Bunodosoma grande* with high support and *Haloclava* sp. is sister to a clade containing *Isosicyonis*, *Glyphoperidium* Roule, 1909 [49], and *Isotealia* Carlgren 1899 [7], but this relationship resolves with low support. *Anemonactis minuta* is in a polytomy with species of *Epiactis* Verrill, 1869 [50] and *Urticina* Ehrenberg, 1834 [60]. Clades 2 and 3 each have maximum support. Relationships within Clade 3 are the same as in the likelihood analysis of the all-loci dataset.

The tree with the highest posterior probabilities for our mitochondrial dataset resolves Haloclavidae into the same three clades seen in the likelihood analysis of the all-loci and mitochondrial datasets. In contrast, the tree with the highest posterior probabilities for our nuclear dataset fails to resolve families and genera, with much of the tree being a polytomy. *Haloclava producta* is well-supported as sister to *Bunodosoma grande*. *Haloclava* sp. is weakly supported as the sister to a clade with *Isosicyonis alba*, *Glyphoperidium bursa*, and *Is*. *striata* Rodríguez & López-González, 2008 [61]. *Harenactis* and *Stephanthus* are not sister taxa in this tree: the position of *Harenacti*s is unresolved and *Stepthanthus* is sister to *Epiactis georgiana*. *Peachia cylindrica* is sister to *Jasonactis erythraios* and *P*. *quinquecapitata* is not resolved. *Synpeachia temasek* is sister to the *Metapeachia tropica* samples, but this is only weakly supported.

### Parsimony analyses

The parsimony analysis of the all-loci dataset yielded 11 equally parsimonious trees. Their strict consensus tree was 19367 steps long, with 2595 parsimony informative sites, CI = 0.352, RI = 0.683. In that tree, Haloclavidae is split into several groups, only one of which (Clade 2) is found in the model-based analysis of the all-loci dataset. In the parsimony consensus tree, *Antennapeachia jambio*, *Anemonactis minuta*, and *Haloclava producta* form a consistent but unsupported clade with *Bunodosoma* as its sister. *Haloclava* is sister to *Phlyctenactis* Stuckey, 1909 [62], but this clade also is not supported in our bootstrap analysis. As in most other analyses (Table 1), *Stephanthus* and *Harenactis* resolve as sister taxa with high bootstrap support. The majority of “*Peachia-*like” species (Clade 3) resolve as a clade with bootstrap support, with *Peachia cylindrica* + *P*. *quinquecapitata* and *Synpeachia temasek* + *Metapeachia tropica* forming well supported subgroups within the larger clade.

The parsimony analysis of the mitochondrial dataset yielded 56 equally parsimonious trees. The resulting strict consensus tree was 4835 steps long, with 826 parsimony informative sites CI = 0.351, RI = 0.815. The strict consensus includes Clades 1, 2, and 3, each of which relates to the remaining Actinioidea in ways that differ from the results based on other datasets and methods. *Haloclava producta* is not within any of the well-supported clades. Clade 1 is sister to a larger group containing all Clade 3, but the relationships are generally not supported. Within Clade 3, *Antennapeachia jambio* is sister to the same *Peachia* clade found in the all-loci analysis. *Peachia cylindrica* and *P*. *quinquecapitata* are sister to each other and this clade is sister to a polytomy containing *Metapeachia tropica* and *Synpeachia temasek*. The parsimony analysis of the nuclear dataset generated 42 trees whose strict consensus tree contained 14087 steps, CI = 0.367, RI = 0.615. The genera of Haloclavidae did not split into the same clades seen in the other parsimony or model-based analyses. *Haloclava* sp. resolved as sister to a species of *Anthopleura*, with low support. *Haloclava producta* is sister to *Bunodosoma grande* with strong support; this clade is in an unsupported polytomy with *Antennapeachia jambio*. *Harenactis* is sister to the *Antennapeachia*/*Ha*. *producta* clade, but this relationship has no support. *Stephanthus* resolved as sister to *Epiactis*. *Peachia cylindrica* and *P*. *quinquecapitata* are not resolved as sister taxa. *Synpeachia* and *Metapeachia* resolve as sister taxa with moderate bootstrap support.

### Ancestral state reconstruction

**Fig 2:**
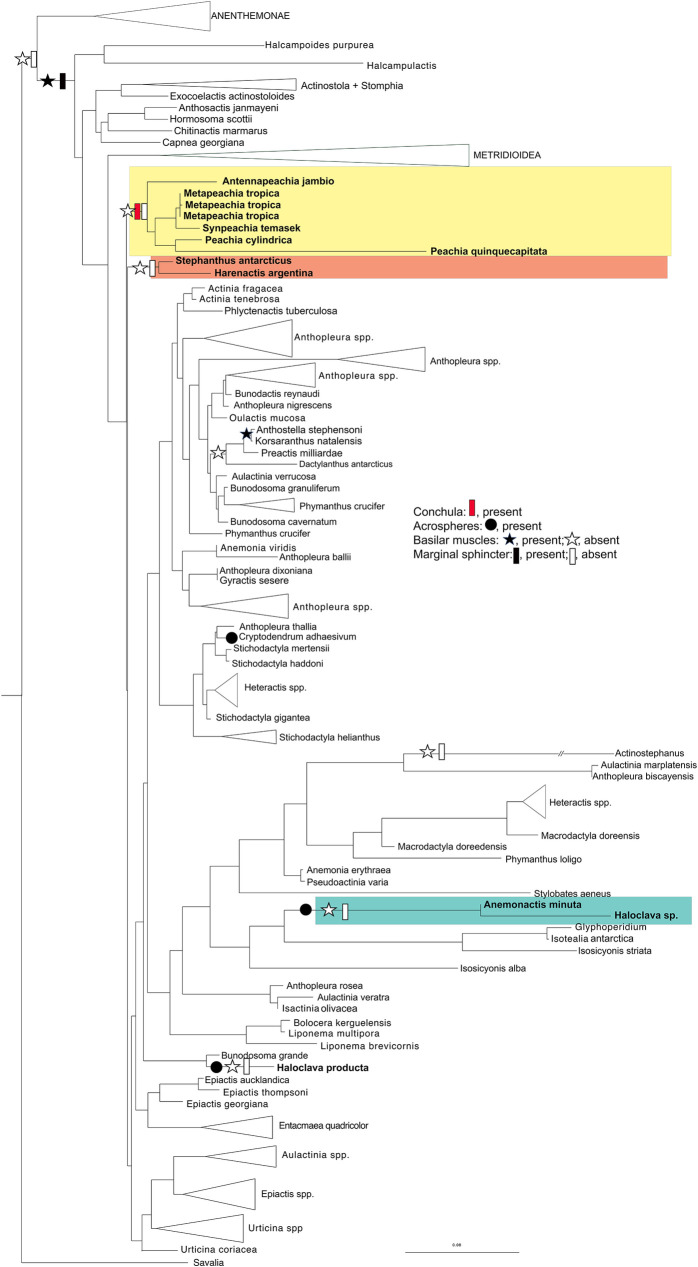
Ancestral state reconstruction of morphological characters. Representation of ancestral character state reconstruction for four morphological characters (conchula, acrospheres, basilar muscles, and marginal sphincter). Characters mapped onto the Maximum Likelihood (ML) analysis of all-loci. Support values are not included for legibility; refer to Fig 1 for values.

The results of the character trait analysis are represented on the tree in Fig 2. The main characters of interest for Haloclavidae are acrospheres and the conchula, as other analyses have shown the basilar muscles and marginal sphincter to be convergent traits at a broad scale [5]. The conchula has evolved once over our tree, in Clade 3, which contains all of the *“Peachia-like”* sea anemones. Acrospheres have evolved twice within Haloclavidae but are seen in two other species on the tree (four total independent evolutions of acrospheres, in the *Haloclava/Anemonactis* clade, and in *Telmatactis* Gravier, 1916 [63] and *Cryptodendrum* Klunzinger, 1877 [64]).

The evolution of musculature in the various clades of Haloclavidae is complex. The inferred ancestral state for Actinioidea is the absence of a marginal sphincter: the endodermal sphincter conventionally ascribed to Actinioidea is inferred under likelihood criteria to have evolved once within this lineage. In Clades 2 and 3, a lack of a marginal sphincter is the inferred ancestral state. In Clade 1, the common ancestor of *Anemonactis* and *Haloclava* is inferred to have lost the sphincter because this clade nests within a clade of actinioideans that has an endodermal marginal sphincter. The lack of basilar muscles in Clades 1, 2, and 3 is inferred under likelihood criteria to be a secondary loss.

### Taxonomic account

Superfamily **Actinioidea** Rafinesque, 1815
Family **Peachiidae fam. nov.** Hamilton, Daly, & Rodríguez, 2022
Genus ***Peachia*** Gosse, 1855
*Peachia chilensis* Carlgren, 1931
*Diagnosis* (after Carlgren (1931), modifications in italics).

**Fig 3:**
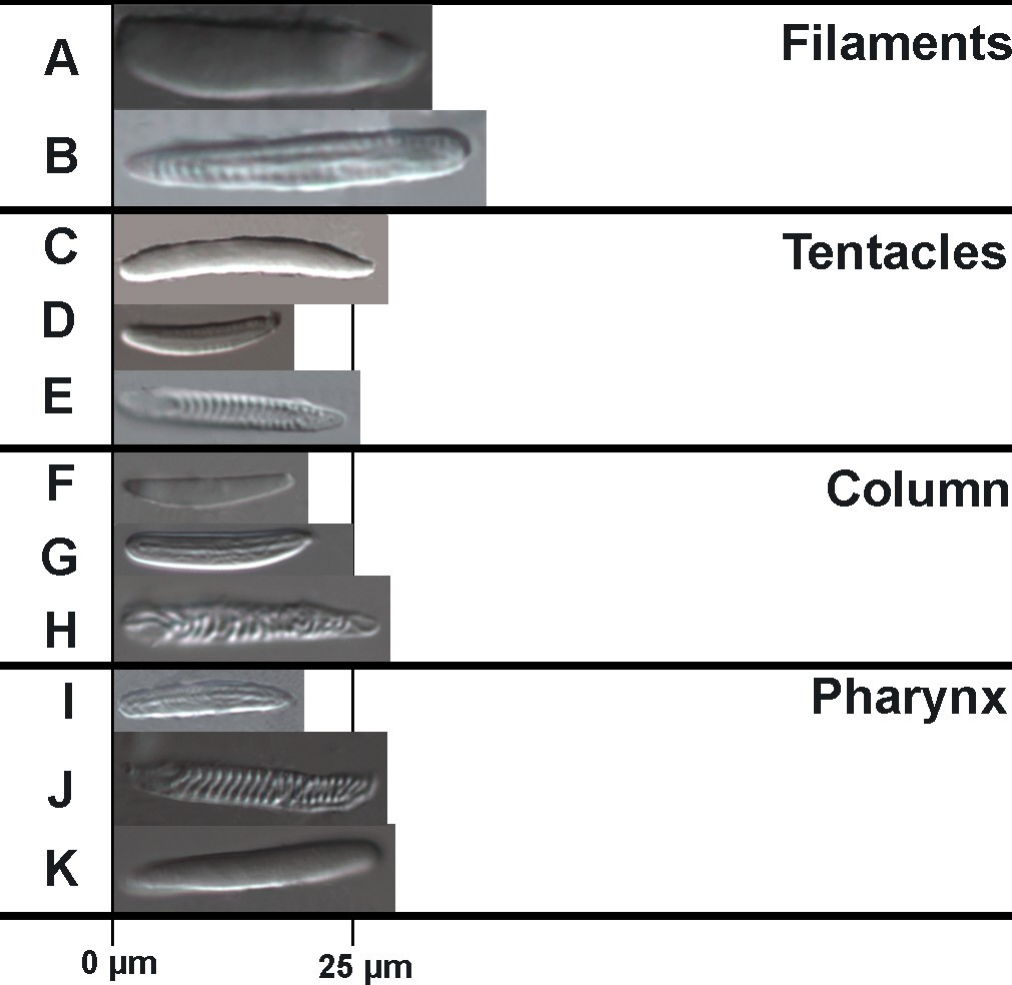
Cnidae of *Peachia chilensis*. Spirocysts: in tentacles (E), column (H), and actinopharynx (J); basitrichs: in tentacles (D), column (G), actinopharynx (I), and mesenterial filaments (B); holotrichs; in tentacles (C), column (F), actinopharynx (K), and filaments (A). See Table 2 for size and distribution.

Elongate Peachiidae, with rounded aboral end. *Known only from juvenile specimens*. Column *smooth* with *longitudinal* rows of cinclides. Marginal sphincter *absent*. Tentacles 12, equal in length. Single *siphonoglyph* with *poorly developed* conchula. All 12 mesenteries perfect. Retractor muscles broad. Basilar muscles absent. Cnidom (Table 2 and Fig 3): *spirocysts*, *basitrichs*, and *holotrichs*.

**Table 2:** Size and distribution of cnidae of *Peachia chilensis*.

Tissue/cnida type	Capsule length x width (μm)	Mean ± SD	N
TENTACLES	(17–23 x 1.5–2)		
Spirocysts (E)	12.52–27.66 x 2.91–5.01	22.01 ± 3.17 x 3.81 ± 0.43	52
Basitrichs (D)	15.39–24.60 x 3.15–4.57	19.14 ± 2.57 x 3.79 ± 0.40	21
Holotrichs (C)	13.88–27.32 x 3.40–4.18	19.19 ± 5.64 x 3.9 ± 0.34	6
COLUMN	(No data)		
Spirocysts (H)	18.06–26.95 x 3.5–5.19	22.50 ± 2.35 x 4.25 ± 0.40	28
Basitrichs (G)	14.19–22.04 x 2.85–5.57	18.69 ± 1.34 x 3.82 ± 0.40	121
Holotrichs (F)	14.23–26.26 x 3.18–4.01	19.15 ± 3.68 x 3.61 ± 0.31	7
PHARYNX	(19–27 x 2–2.5)		
Spirocysts (J)	17.45–24.92 x 3.49–4.09	20.36 ± 3.20 x 3.76 ± 0.26	4
Basitrichs (I)	14.45–27.07 x 3.06–5.07	19.65 ± 3.78 x 3.89 ± 0.45	50
Holotrichs (K)	22.81–27.35 x 4.12–4.31	25.80 ± 3.21 x 4.21 ± 0.13	2
FILAMENTS	(No data)		
Basitrichs (B)	20.62–28.48 x 3.47–5.19	25.20 ± 2.81 x 4.41 ± 0.64	8
Holotrichs (A)	18.35–30.85 x 4.16–4.34	24.60 ± 6.25 x 4.25 ± 0.09	2

**Fig 4:**
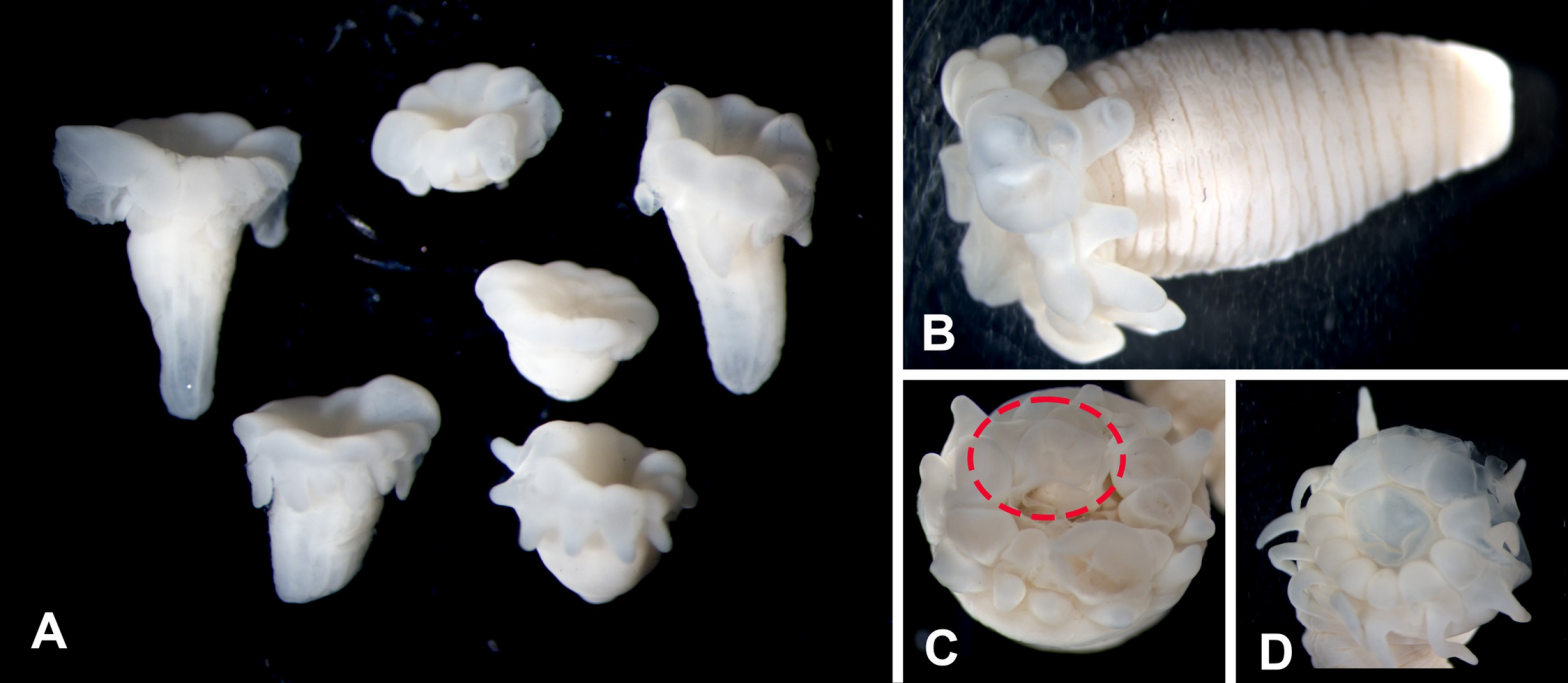
External anatomy of *Peachia chilensis*. (A) Lateral view of six of the smaller specimens of *P*. *chilensis*, showing the diversity in developmental stages in the smallest of the specimens. (B) Lateral view of larger specimen showing the lack of visible capitulum, scapus, or physa. (C) Dorsal view of specimen showing the protruding conchula (circled in red). (D) Dorsal view of specimen, showing esophageal folds.

Letter after each type of capsule refers to Fig 4. SD = Standard Deviation, N = Number of capsules measured. Measurements from Carlgren’s (1931) original description in parentheses.

**Fig 5:**
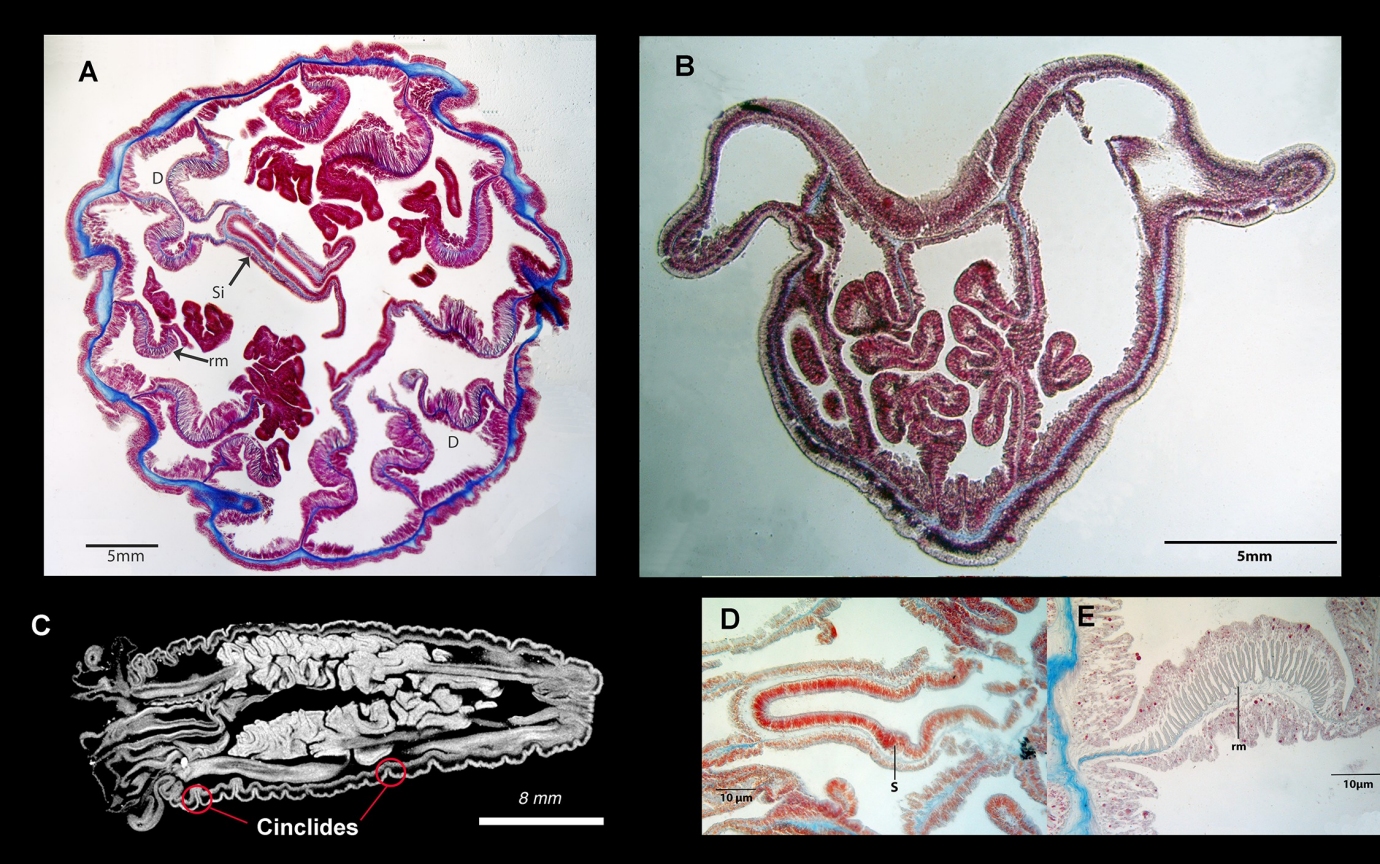
Internal anatomy of *Peachia chilensis*. (A) Histological cross section through the column showing the cycles of the mesenteries and the siphonoglyph not completely separated from the actinopharynx. Mesentery pairs numbered. (B) Histological longitudinal section through the entire animal, highlighting the absence of a marginal sphincter. (C) Longitudinal micro-CT scan with the cinclides visible. (D) Histological cross section highlighting the siphonoglyph. (E) Cross section highlighting the detail of a retractor muscle. D = directives, si = siphonoglyph, rm = retractor muscle.

External anatomy: Body vermiform, without distinguishable scapus or capitulum. Column narrower at aboral end and wider at oral end, without distinguishable physa (Fig 4A and 4B). Preserved specimens to 14 mm length and 5 mm diameter. Oral disc relatively small (Fig 4C and 4D), with esophageal lobes associated with each tentacle (Fig 4D). Tentacles 12, equal in length, arranged in single cycle, tapering, with cnidae distributed over entire surface. Tentacles without acrospheres. Distal part of siphonoglyph extends onto oral disc as a conchula (Fig 4B). Lobes on conchula variable in number across examined specimens, absent in smallest specimens. Longitudinal rows of cinclides detected in CT scans (Fig 5C) but not readily distinguished otherwise.

Internal anatomy: Single, prolonged, deep siphonoglyph (Fig 5D). All mesenteries perfect (Fig 5A). Number of mesenteries 12, identical at proximal and distal ends. Marginal sphincter absent (Fig 5B). Two directive pairs, one attached to well-developed siphonoglyph (Fig 5A); other opposite it, attached to unremarkable portion of actinopharynx (Fig 5A). Retractor muscles broad and comb-like, with many folds all on one surface of mesentery (Fig 5E), similar in size and development distally and proximally. Parietal muscles not distinguishable, inferred to be very weak, if present.

## Discussion

### Phylogeny and diversity of Haloclavidae

All previous analyses have revealed that burrowing sea anemones do not represent a clade, with basilar musculature inferred to have been repeatedly lost across Actiniaria [4, 31] because burrowing anemones nest within groups whose members largely have basilar muscles and live attached to the substrate. These large-scale phylogenetic analyses have also revealed that Haloclavidae is not monophyletic. Daly et al. [5] found *Peachia cylindrica*, *Stephanthus antarcticus*, and *Harenactis argentina* in the same clade, distinct from and only distantly related to *Haloclava*. Our results align with these previous phylogenetic analyses in that burrowing sea anemones, and most notably Haloclavidae, are polyphyletic, however, we find different resolutions and different levels of support across datasets and analytical methods. Although we recognize that some parameter set may exist under which Haloclavidae is monophyletic, there is no support for a monophyletic Haloclavidae across methods (Table 1) or in previous studies [4, 5, 31], therefore we do not consider the non-monophyly in our result indicative of a methodological problem.

We find three major clades of Haloclavidae, identified as Clades 1, 2, and 3 (Fig 1). These clades are fairly consistent, generally occurring in the analyses of the all-loci and mitochondrial data sets. Clade 1 consists of *Anemonactis minuta*, *Haloclava producta*, and *Haloclava* sp. Because previous datasets did not include any species of *Anemonactis*, this finding is new, but it reflects long-recognized similarities between these genera [see, e.g., [65]]. Inclusion of *Ha*. *producta* in Clade 1 is more dataset and analysis dependent than the grouping of *A*. *minuta* with *Haloclava* sp. (Table 1). Clade 2, which contains *H*. *argentina* and *S*. *antarcticus* as sister taxa, has been seen in earlier studies [4, 5] and is often associated with or sister to species of *Urticina* or *Epiactis*. We find with consistent and strong support that *Peachia cylindrica* belongs in a clade with other “*Peachia*-like” sea anemones and that this clade (Clade 3) is sister to a large group of Actinioidea, which includes other haloclavids. This differs from previous phylogenetic analyses (i.e. [5]) in which *Peachia* is sister to the haloclavids *Stephanthus* and *Harenactis*. This difference in tree topology is likely due to the broader sampling of taxa in our study compared to previous phylogenetic analyses. Although we have not jackknifed our dataset to identify which specific taxon or combination of taxa are responsible for the change in topology, a deep body of empirical and modeling studies show a positive correlation between increased taxa sampling and increased stability/accuracy of phylogenetic results [66–68].

Our results are largely consistent across datasets and analytical methods (Table 1), however, there is a large difference in the topology and resolution of the three clades between the nuclear dataset and the all-loci or mitochondrial datasets. Such discrepancies between nuclear and mitochondrial phylogenies have been noted in Anthozoan groups [11 and references within]. The nuclear datasets fail to resolve superfamilies, families, or genera that are well-supported in other analyses (such as those seen in [4, 5, 31]). In the analyses of nuclear datasets (and maximum parsimony, MP, analysis of the all-loci dataset), *Antennapeachia jambio*, *Haloclava* sp., and *Bunodosoma granuliferum* [69] form a clade. It is possible that *An*. *jambio* does not resolve with the other *Peachia* anemones in the results based on nuclear data alone because it has a relatively long branch in the nuclear analyses. *Haloclava* sp. also fails to fall within a clade of haloclavids in the nuclear analysis (as well as MP and Bayesian analyses of the all-loci dataset). *Haloclava* sp. has a particularly long branch when compared to the nuclear tree and the mitochondrial data for the same species and typically groups with other species that have long branches but are not historically phylogenetic or taxonomic groups. Additionally, the lack of resolution of Clades 1, 2, and 3 could potentially be due to the variability in length of the nuclear markers. 18S rDNA and 28S rDNA are long sequences that were split into three and five different fragments, respectively. Not all species have the complete sequences for each fragment. Despite some inconsistencies in the nuclear data, the results consistently demonstrate the inadequacy of the current family-level grouping of Haloclavidae and highlight lineages and character systems that require further evaluation.

The trees recovered in our analyses and the accompanying character analyses affirm the interpretation that obligate burrowing lifestyles have evolved repeatedly accompanied by loss of basilar muscles (and often marginal muscles). This pattern has become more apparent as additional burrowing lineages have been described or studied [4–6, 10, 70] and is robust across data type [11], contradicting recent contentions (Ivanova, 2020 [71]) that the monophyly of “Athenaria” remains an open question. The present results, and those of previous studies [5, 8, 9, 11, 31, 72], which find burrowing anemones previously grouped in Athenaria dispersed across the tree, disagree with Ivanova’s suggestion that “Athenaria” should be maintained as a subordinal classification. Over-emphasis on burrowing and characteristics correlated with it obscures similarities and differences in other aspects of anatomy or cnidae and preserves an unnatural and ultimately confusing classification.

### New higher-level classifications

Across analytic methods, we consistently find Clades 1, 2, and 3 with the same members across markers and analyses. This consistency supports the splitting of Haloclavidae into three separate families: Haloclavidae (Clade 1), Harenactidae fam. nov. (Clade 2), and Peachiidae fam. nov. (Clade 3). Diagnostic anatomical and cnida features and constituents of each group are listed in the S1 Appendix. Genera included in Haloclavidae were initially added due to their general morphological similarities; elongated bodies, round aboral end, general lack of basilar musculature. These attributes are common among burrowing anemones. The single, strong siphonoglyph does not unite the focal genera of our study but appears to be widely occurring across Actinioidea. Our findings based on DNA evidence do however correspond with some morphological trends, including the acrospheres, conchula, and cnidom and show some biogeographic consistency. Acrospheres have evolved three times over our tree and the conchula has evolved once (Fig 2). The conchula can also be found in *Actinoporus*, which is not included in our analyses. Because we were unable to include members from Mesacmaea Andres, 1883 [73] or *Tenactis*, we have placed these genera in their respective families based on morphological characteristics.

After the creation of the two new families, Haloclavidae consists of three genera; *Anemonactis*, *Haloclava*, and *Mesacmaea*. This family retains the original description of Haloclavidae, details of which are in the S1 Appendix. In our analysis, we include three members of this family (*As*. *minuta*, *Haloclava* sp. and *Ha*. *producta*) and find consistent but weak support for their grouping (as Clade 1: see Figs 1 and 2). The genus *Haloclava* contains six accepted species; *Haloclava brevicornis* (Stimpson, 1856) [28], *Haloclava capensis* (Verrill, 1865) [74], *Halolcava chinensis* Carlgren, 1931 [75], *Haloclava hercules* Izumi, 2021 [76], *Haloclava producta*, and *Haloclava stimpsonii* (Verrill, 1868) [77]. Species in this genus have 20 tentacles, acrospheres, and extremely long basitrichs [78]. *Anemonactis* has four accepted species [17]; *Anemonactis clavus* (Quoy & Gaimard 1833) [79], *Anemonactis mazeli* (Jourdan, 1880) [80],the recently revised Japanese species *Anemonactis minuta* (Wassilieff, 1908) [48] and *Anemonactis tohrui* Izumi, Yanagi & Fujita, 2020 [65]. It also has one valid but dubious species, *Anemonactis globulosa* Quoy and Gaimard, 1833 [79] (see [3, 65]). *Anemonactis* is similar to *Haloclava* in lacking a conchula and having acrospheres; additionally, its members have 12–20 tentacles and 20 longitudinal rows of cinclides, lack *p-*mastigophores, and have very long basitrichs [65]. *Anemonactis minuta* was until recently reported to have a very broad range, with the name in use for specimens from Japan as well as Europe until Izumi et al. [65] clarified the different identity of the Japanese species within *Anemonactis*. The type species of *Anemonactis*, *As*. *clavus*, was originally reported as parasitic as a larva, with 12 tentacles and a conchula, and this species possibly aligns with *Peachia*, rather than with *As*. *minuta* and *Haloclava* in Haloclavidae. Future molecular and morphological work would determine the correct placement of this species; recognizing its status as the type of the genus, we prefer to wait until a thorough revision of material is done to make any taxonomic changes.

The genus *Mesacmaea* remains in the family Haloclavidae currently, but its taxonomic assignment could very well change when they are included in a molecular phylogenetic analysis. *Mesacmaea* has three valid species; *Mesacmaea chloropsis* (Agassiz in Verrill, 1864 [81]), *Mesacmaea laevis* (Verrill, 1864 [81]), and *Mesacmaea mitchelli* (Gosse, 1853) [78]. Members of this genus have up to 36 tentacles, 18 mesenteries and a diffuse mesogleal marginal sphincter. Although it is possible that an anemone with a marginal sphincter could exist within a group of burrowing sea anemones (which generally lack marginal sphincters because their bodies are narrow and vermiform), this is one of the only species of Haloclavidae that has a marginal sphincter (the other being *Tenactis riosmenai* Barragán, Sánchez & Rodríguez, 2018 [20], discussed below). Other members of Actinioidea either have an endodermal sphincter muscle or lack this muscle, so we expect *Mesacmaea* may not fall into Haloclavidae or even within Actinioidea if a molecular analysis were conducted. In addition, the mesentery arrangement of *Mesacmaea–*in which all mesenteries are perfect, arranged bilaterally, with pairs of mesenteries after the first 10 pairs (first and second cycles) developed only in the middle exocoels and not in the lateral ones [22], more closely resembles some members of the family Actinernidae than any other actinioidean. However, until a detailed revision is finished, we prefer to leave *Mesacmaea* within Haloclavidae.

The family Harenactidae (Clade 2 in our analyses) consists of the genera *Harenactis* and *Stephanthus*. We create this family due to the consistent grouping of these genera in our molecular analysis and in other phylogenetic studies [4, 5, 31]. The members of this family are morphologically differentiated from other species in the former Haloclavidae because they have a well-developed fosse and parapet and 12 pairs of mesenteries (two complete cycles, i.e. 6+6 pairs). There are no other clear anatomical or microanatomical features that group these species, despite strong molecular evidence for their close relationship. The genus *Harenactis* consists of two species: *Harenactis attenuata* Torrey, 1902 [19] and *Harenactis argentina*. Members of this genus have 24 tentacles, 12 pairs of perfect mesenteries, and distal cinclides; they lack a conchula. *Stephanthus* is monotypic. Its sole species, *S*. *antarcticus*, was placed into the family Haloclavidae due to a single, very strong siphonoglyph and lack of basilar musculature and *p*-mastigophores; the last of these features resembles *Anemonactis*. The two genera of this family can be distinguished from each other as *Harenactis* has only perfect mesenteries, reniform retractor muscles, and cinclides while *Stephanthus* has perfect and imperfect mesenteries, diffuse retractor muscles, and no cinclides. This family adopts the name Harenactidae as *Harenactis* was the first genus of this family to be described.

The family Peachiidae (Clade 3 in our analyses) consists of the genera *Antennapeachia*, *Metapeachia*, *Peachia*, *Synpeachia*, and *Tenactis*. We create this family due to the support of Clade 3 across our molecular analyses and in recognition of the morphological similarities that have been noted in the description and naming of these taxa. This family adopts the name Peachiidae as *Peachia* is the oldest genus and the point of comparison for the description of the others. It is characterized by the shared occurrence of the conchula.

*Peachia* is the oldest and most species-rich and oldest genus in this group, being described in 1855 and containing 11 species; *Peachia carnea* Hutton, 1880 [82]; *Peachia chilensis*; *Peachia cylindrica*; *Peachia hastata*; *Peachia hilli*; *Peachia koreni* McMurrich, 1893 [83]; *Peachia mira* Carlgren, 1943 [24]; *Peachia neozealanica* Carlgren, 1924 [84]; *Peachia parasitica*; *Peachia quinquecapitata*; and *Peachia taeniata* Klunzinger, 1877 [64]. Members of this genus are burrowing anemones with 12 tentacles and all are thought to be parasitic during their larval stage. Differences between species of this genus are not yet well understood, with geographic location and size differences being the main features to distinguish species. Historically, conchula morphology was used to determine species, but this trait has been shown to be highly variable depending on organism age and fixation [11].

*Antennapeachia* contains two valid species; *Antennapeachia jambio* and *Antennapeachia setouchi* Izumi, Yanagi & Fujita, 2016 [26]. Both of these are known only from Japan. Species within *Antennapeachia* are differentiated from each other by the number of marginal tentacles, mesentery arrangement, and the cnidom. They are differentiated from other members of the family as they have six pairs of macrocnemes and two pairs of one microcneme and one macrocneme, with 12 regular tentacles and two irregular tentacles that are raised upward, like antennae. The role of these tentacles is not yet known, but it is speculated that they might be functionally similar to marginal tentacles [51]. Also the cnidom of this genus is remarkable within the family (Table 3). The genus *Metapeachia* has two species; *Metapeachia tropica* and *Metapeachia schlenzae* Gusmão, 2016 [9]. The type species, *M*. *tropica*, was originally placed in the genus *Peachia* [52] but Carlgren [24] created a new genus for it because it had a different number of tentacles and mesenteries and had a siphonoglyph completely separated from the actinopharynx. The eight perfect mesenteries and tentacle number distinguish *Metapeachia* from all other members of Peachiidae (Table 3). Differences between *M*. *tropica* and *M*. *schlenzae* are microanatomical [9]. *Synpeachia* contains only *Synpeachia temasek*. This species has a siphonoglyph completely separated from the actinopharynx but is distinguished from *Metapeachia* by its number of tentacles, cnidae, and lack of cinclides. The phylogenetic results underscore the similarities between *Synpeachia* and *Metapeachia*, as they are well supported as sisters, with *Metapeachia* paraphyletic with respect to *Synpeachia* in these analyses.

**Table 3:** Morphological differences in species with conchula previously placed in Haloclavidae (Verrill 1899).

Genus	Tentacle Number	Mesenteries	Conchula	Marginal sphincter	Siphonoglyph	Acrospheres	Cinclides	Fosse and parapet	Column	Cnidom
*Anemonactis*	12–20	6 pairs perfect, 4 pairs imperfect	Mostly absent	Absent	Single, strong	Present	Present, 20 longitudinal rows in distal part of column and proximal end	Absent	Not divisible into regions, with numerous solid papillae	Basitrichs, *b*-mastigophores, spirocysts
*Antennapeachia*	14 or 16	8 pairs perfect, 2 independent imperfect	Present	Absent	Single, deep, never forming distinct tube separated from actinopharynx	Absent	Absent	Absent	Not divisible into regions, minute adherent areas, aboral end rounded	Basitrichs, *b*-mastigophores,*p*-mastigophores A, A with looped proximal tubule, B1, B2a, spirocysts
*Metapeachia*	16	8 pairs perfect	Present	Absent	Separated from the actinopharynx only by a small strip of tissue, forms a distinct tube	Absent	Present, 16 longitudinal rows in distal part of column	Absent	Not divisible into regions, sticky in live specimens, proximal end physa-like	Basitrichs, *b*-mastigophores,*p*-mastigophores A, spirocysts
*Peachia*	12	6 pairs perfect, 4 pairs imperfect	Present	Absent	Rarely separated from actinopharynx	Absent	Present, longitudinal rows in the proximal end of physa-like sructure	Absent	Not divisible into regions, minute adherent areas	Basitrichs, holotrichs, spirocysts
*Synpeachia*	20	10 pairs perfect	Present, 5-lobed	Absent	Single, deep, completely separated from the actinopharynx, forms a distinct tube	Absent	Absent	Absent	Not divisible into regions, sticky in live specimens, proximal end physa-like	Basitrichs, *b*-mastigophores,*p*-mastigophores A, spirocysts
*Tenactis*	20	10 pairs perfect	Poorly developed	Endodermal, weak and diffuse	Single, strong	Absent	Present	Fosse present	Not divisible into regions, with verrucae	Basitrichs, *b*-mastigophores, *p*-mastigophores A, A with looped proximal tubule, B1 and B2a, spirocysts

Characteristics of genera in Peachiidae (Modified from [9]).

The monotypic genus *Tenactis* is the most recent genus to be added to the family Haloclavidae and its addition to the family necessitated revision of the diagnosis of the family. *Tenactis riosmenai* is the only haloclavid with a pedal disc with basilar muscles, diffuse endodermal marginal sphincter muscle, pseudo-acrorhagi, a single pair of directives, adherent verrucae, and *p*-mastigophores B1. This species was placed in the family Haloclavidae due to it having a single, strong siphonoglyph, only 10 pairs of perfect mesenteries, cinclides at the base, and a poorly developed conchula. This mesentery arrangement, presence of cinclides, and conchula are now characteristics that that characterize the newly erected family Peachiidae. In addition, *Tenactis* shares similarities in the cnidae with the species of *Antennapeachia* having *p*-mastiogphores B2a and *p*-mastigophores A with a looped proximal tubule in the filaments.

### Identity of *Peachia chilensis*

*Peachia chilensis* has only ever been reported in its larval form and has not been well documented in the literature. Riascos et al. [85] describe the association between *P*. *chilensis* and the scyphomedusa *Chrysaora plocamia* [86], but provide no description of the sea anemone, include only one picture of it within the text, and do not mention voucher specimens. The only other records of *P*. *chilensis* in the literature are reviews that document anthozoan biodiversity [3, 87], and these, like the account of Riascos et al. [85] do not provide new details or specimens. Puente-Tapia et al. (2021 [88]) give a detailed description of an Argentinian *Peachia* sp. at parasitic and free-living life-stages, but do not identify this as *P*. *chilensis*. A molecular study is necessary to determine if their *Peachia* sp. is the same as what we have described here.

The specimens we examined have relatively short tentacles, a vermiform shape, a single deep siphonoglyph, and lack basilar muscles, acontia, and marginal sphincter muscle. They have a weak conchula. In addition to the four genera with conchula previously mentioned (*Antennapeachia*, *Metapeachia*, *Peachia* and *Synpeachia*), *Tenactis* and *Anemonactis clavus* (in some descriptions) also have a poorly-developed conchula that consists of a small, tongue-like outgrowth at the entrance of the siphonoglyph [20, 89]. The prominent cinclides and number of mesenteries in these specimens align with *Peachia* rather than any of the other conchula-bearing genera (Table 3). Additionally, our specimens were discovered parasitizing a jellyfish, a characteristic unique to *Peachia* (plus *An*. *clavus* in some descriptions) among haloclavid anemones. The weakness of the conchula, compared to what is expected of adult members of *Peachia*, may reflect variation due to age and size of our specimens, or *P*. *chilensis* may be a species of *Peachia* that has a relatively weak conchula.

All other described species within the genus *Peachia* have 10 pairs of mesenteries (6+4), while our samples have six pairs (6+0). This likely reflects the developmental stage of the anemones [90]: our specimens are presumably in their larval state, given their size and that they were found parasitizing jellyfish. Other reports of larval *Peachia* record only six pairs of mesenteries, with the four imperfect mesenteries not yet developed [24, 32]. The larval form of *P*. has also been described as having 12 mesenteries, but four of them do not reach the actinopharynx [91].

Of the 11 species currently reported in *Peachia*, four are known from both the larvae and adults (*P*. *hilli*, *P*. *quinquecapitata*, *P*. *parasitica*, and *P*. *hastata*). Based on these, we can compare the parasitic and free-living forms. For example, tentacle number is generally consistent between larval sea anemones and adults, with the 12 tentacles of the larval anemone being shorter [42, 43]. The conchula is more pronounced in adults [9, 22]. Based on what is known of from the species for which both larvae and adults are known, we expect the adult form of *P*. *chilensis* to have (6+4) pairs of mesenteries, a more pronounced conchula, and be a sessile, burrowing animal [91].

Seven species of burrowing anemones that lack a pedal-disc have been reported from Chile [68]: *Scolanthus intermedius* (McMurrich, 1893) [83] and *Edwardsiella ignota* (Carlgren 1959) [92] within Edwardsiidae Andres, 1881 [12]; *Galatheanthemum profundale* Carlgren, 1956 [93] within Galatheanthemidae Carlgren, 1956 [93]; *Cactosoma chilense* (McMurrich, 1904) [94] and *Halcampa abtaoensis* Carlgren, 1959 [92] within Halcampidae Andres, 1883 [73], *Octineon chilense* Carlgren, 1959 [92] within Octineonidae Fowler, 1894 [95]; and *Peachia chilensis* within Haloclavidae. *Scytophorus striatus* Hertwig, 1882 [1], within the recently resurrected Halcampoididae Appellöf, 1896 [96] (see [97]) is reported also in Chile [87], but this record is not validated here [97]. Although several of these species have not been seen since their original description or have only been reported from deep waters, we compare and distinguish them from the specimens of *P*. *chilensis* redescribed here. Among the burrowing anemones reported from the region, members of Halcampoididae are most likely to be mistaken with members of *Peachia* because both lack basal and marginal musculature and several genera and species in Halcampoididae have 12 mesenteries. However, the actinopharynx of *Scytophorus* (and other members of Halcampoididae) lacks a conchula [97].

*Peachia chilensis* differs from the other Chilean burrowing anemones based on family- and genus-level characteristics. They do not fit the diagnosis of Edwardsiidae (*Sc*. *intermedius* and *E*. *ignota*) because members of Edwardsiidae have only eight perfect mesenteries as adults whereas our larval specimens have 6 pairs of perfect mesenteries (i.e. 12 mesenteries). *Galatheanthemum profundale* is a deep-sea species, and its members have a tube-like cuticle and a mesogleal marginal sphincter; our specimens have no cuticle or marginal sphincter. *Cactosoma chilense* is described as having a mesogleal marginal sphincter and a body divisible into capitulum, scapus, and physa–all of these characteristics differ from those seen in our specimens. *Halcampa abatoensis* is described as having 8–10 tentacles, unlike the 12 in *P*. *chilensis*. *Octineon chilense* has an adherent base, a strong mesogleal sphincter and acontia whereas the specimens described in this study do not. *Scytophorus striatus* is also a deep-sea species that has 14 mesenteries and 14 tentacles and a shallow siphonoglyph whereas *P*. *chilensis* has 12 mesenteries and tentacles and a strong siphonoglyph.

## Supporting information

S1 Table Taxa included in this study. New sequences are indicated by bolded Genbank accession numbers.
(XLSX)

S2 Table Parameters implemented within MrBayes v.3.2.7. Mt: mitochondrial. Nc: nuclear. #: number. All analyses utilized 2 runs, 4 chains, and had a sample frequency of 100. Model of nucleotide substitution: GTR+I+G (COIII, 12S, 16S, 28S) and SYM+I+G (18S).
(XLSX)

S3 Table Character state matrix. 0 = character absent, 1 = character present,? = unknown.
(XLSX)

S4 Table Results from parsimony analyses of each data set. (XLSX)

S1 Appendix Taxonomic results. (DOCX)
