# Supplementary material for: Phylogeny and taxonomy of Haloclavidae (Verrill, 1899) with a redescription of the parasitic, burrowing sea anemone, Peachia chilensis Carlgren, 1931
Source: PLoS One. 2022 Sep 16;17(9):e0266283. doi: 10.1371/journal.pone.0266283 (PMC9481011; doi:10.1371/journal.pone.0266283)
Supplement: S1 Appendix — (DOCX) [file pone.0266283.s005.docx]

**S1 Appendix: Taxonomic results**

**Taxonomic changes**

We define and list taxonomic changes below, using italics to indicate modifications to cited diagnoses and asterisks to indicate taxa whose membership or taxonomic placement has changed. Underlined taxa are included in molecular analyses (Fig. 1). Type genus of each family indicated by bold font. Cnidom is based on existing reports with the recognition that the current understanding of this attribute is incomplete for many taxa. We erect new taxa based on morphological and our new molecular data. Authorship for new names contained here should be attributed to Hamilton, Daly, and Rodríguez.

**Order Actiniaria Hertwig, 1882**

**Suborder Enthemonae Rodríguez & Daly in Rodríguez et al 2014**

**Superfamily Actinioidea Rafinesque 1815**

*Family Haloclavidae** Verrill 1899

*Diagnosis*(modified after Carlgren, 1949)

Body elongate, without basilar musculature; aboral end generally physa-like, flattened and adherent in *Mesacmaea.* Marginal sphincter absent or mesogleal and diffuse in *Mesacmaea*. Column not divisible into physa, scapus, and scapulus. Column with papillae, with or without cinclides. *Same number of mesenteries distally and proximally*. Perfect pairs of mesenteries few – 8–10 (or 36 in *Mesacmaea*). Two pairs of directives. Single, strong, ventral siphonoglyph, not wholly separated from actinopharynx. Cnidom: spirocysts, basitrichs, and *b*-mastigophores; no *p*-mastigophores except in *Mesacmaea*.

*Etymology*

The name Haloclavidae is derived from the type genus, *Haloclava* Verrill, 1899.

*Included genera*

*Anemonactis* Andres, 1881*,* ***Haloclava* Verrill, 1899**, *Mesacmaea* Andres, 1883 (?).

*Remarks*

The membership of Haloclavidae has been adjudicated based on morphological features. Because molecular evidence does not support a close relationship among several of the genera previously included in Haloclaviade (Fig. 1), we restrict its membership by removing all taxa that have been shown to be only distantly related to the type genus, *Haloclava*. It is possible that some genera not included in the present analysis, specifically *Mesacmaea*, could be more appropriately placed in a different family, such as Actinernidae (uncertainty indicated by a question mark). We have modified the number of mesenteries following the revision of the Japanese *Anemonactis* spp. by Izumi et al. [65] and added information about mesenteries distally and proximally.

*Family Harenactidae fam. nov.*Hamilton, Daly, & Rodríguez, 2022

*Diagnosis*

Body elongate, without basilar musculature; aboral end physa-like, sometimes broadly adherent. Marginal sphincter absent. Column smooth, with or without visible cinclides; sometimes with superficial spots. Same number of mesenteries distally and proximally. Twelve pairs of mesenteries, two pairs directives; first cycle of mesenteries perfect, second cycle perfect or imperfect. Tentacles 24. Single, strong, ventral siphonoglyph, not wholly separated from actinopharynx. Cnidom: spirocysts, basitrichs, *b*-mastigophores and *p*-mastigophores A (latter category absent in *Stephanthus*).

*Etymology*

The name Harenactidae is derived from the type genus, *Harenactis* Torrey, 1902.

*Included genera*

***Harenactis* Torrey, 1902****, Stephanthus* Rodríguez & López-González, 2003*.

*Family Peachiidae fam. nov.*Hamilton, Daly, & Rodríguez, 2022

*Diagnosis*

Body elongate; aboral end physa- or pedal disc-like; with (in *Tenactis*) or without basilar musculature. Marginal sphincter absent or endodermal and diffuse (in *Tenactis*). Column not divisible into physa, scapus, and scapulus; smooth, with or without visible cinclides, sometimes with rows of verrucae. Fosse may be present. Same number of mesenteries distally and proximally. Mesenteries in 6–10 pairs, one or two directive pairs. Tentacles short, 12–20. Single, strong, ventral siphonoglyph, sometimes wholly separated from actinopharynx, with oral end drawn out into lobate conchula. Conchula distinct but varies in shape and number of lobes. Cnidom: spirocysts, basitrichs, holotrichs, *b*-mastigophores, *p*-mastigophores A, B1, and B2a, and *p*-mastigophores A with looped proximal tubule.

*Etymology*

The name Peachiidae is derived from the type genus, *Peachia* Gosse, 1855.

*Included genera*

*Antennapeachia* Izumi, Yanagi, & Fujita 2016**, Metapeachia* Carlgren, 1943**,* ***Peachia* Gosse, 1855****, Synpeachia* Yap, Fautin, Ramos & Tan*, *Tenactis* Barragán, Sánchez & Rodríguez, 2018*.
